
JOURNAL INFORMATION
==============================
NLM Title Abbreviation: City Soc (Wash)
Journal ID: City Soc (Wash)
NLM Journal ID: 101561710
Journal ID: CISO

City & Society (Washington, D.C.)

ISSN: 0893-0465
EISSN: 1548-744X

ARTICLE INFORMATION
==============================
PMCID: PMC7436564
PMID: 32836791
DOI: 10.1111/ciso.12336
Article ID: CISO12336
Article version: 1
Subjects: City & Society, COVID‐19 City Dispatches

Crisis in Göttingen’s Corona High‐Rises: Rethinking Urban Justice amid the Pandemic

Alyanak Oğuz 1
1 Volkswagen Foundation Postdoctoral Fellow at the University of Göttingen, Germany
Electronic publication date: 2020 Aug 12
Print publication date: 2020 Dec
Volume: 32
Issue: 3
Electronic Location ID: 10.1111/ciso.12336
Copyright: © 2020 The Authors. City & Society published by Wiley Periodicals LLC on behalf of American Anthropological Association.
License: This is an open access article under the terms of the http://creativecommons.org/licenses/by/4.0/ License, which permits use, distribution and reproduction in any medium, provided the original work is properly cited.
License URL: https://creativecommons.org/licenses/by/4.0/

Figure count: 0
Table count: 0
Page count: 14

source-schema-version-number: 2.0
cover-date: December 2020
details-of-publishers-convertor: Converter:WILEY_ML3GV2_TO_JATSPMC version:6.1.7 mode:remove_FC converted:13.07.2022

(Correction added on 20 August 2020, after first online publication: Projekt Deal funding statement has been added. Figure placements were updated.)

==============================

CITY & SOCIETY

City & Society

### Crisis in Göttingen's Corona High-Rises: Rethinking Urban Justice amid the Pandemic

**Oğuz Alyanak,** Volkswagen Foundation Postdoctoral Fellow at the University of Göttingen, Germany

On 18 June 2020, the Groner Landstraße 9, a high-rise in the German city of Göttingen, was put under a month-long lockdown after 120 of its residents had tested positive for COVID-19. Two days later, the residents clashed with the police, "throwing stones, tires and a computer," and setting items on fire, "before being forced back with tear gas" (Deutsche Welle 2020).

The night of the clash, I received a call for solidarity with the residents of the high-rise. The call was extended by a group called Anarchistiches aus Göttingen, Anarchists from Göttingen, which, to my knowledge, comprises of several anti-fascist and leftist factions in the city, many of whom gather under the banner of Antifaschistische Aktion, or Antifa.

© 2020 The Authors. City & Society published by Wiley Periodicals LLC on behalf of American Anthropological Association. This is an open access article under the terms of the Creative Commons Attribution License, which permits use, distribution and reproduction in any medium, provided the original work is properly cited. DOI: 10.1111/ciso.12336.
(Correction added on 20 August 2020, after first online publication: Projekt Deal funding statement has been added. Figure placements were updated.)

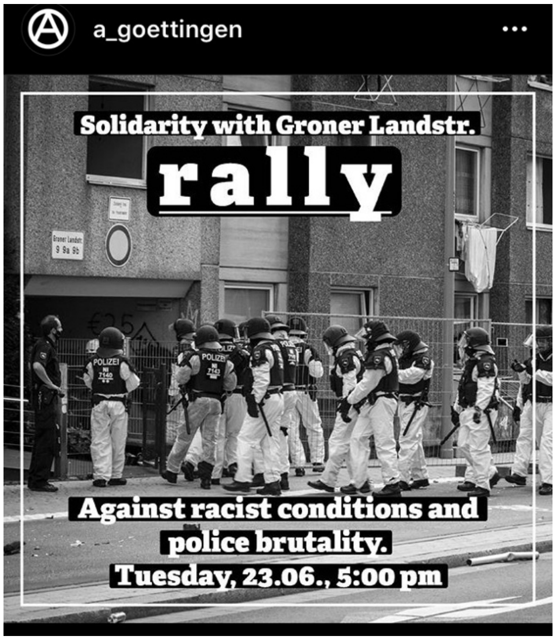

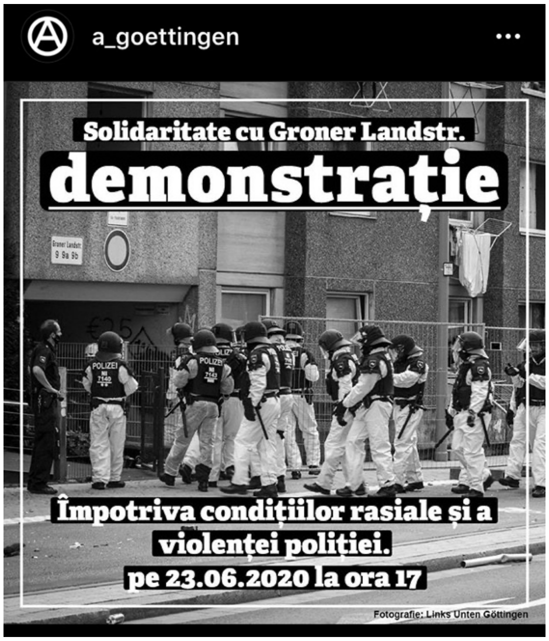

"In Groner Landstraße 9a-c in Göttingen, more than 700 people, including 200 children, have been under quarantine," the organizers wrote in another post, and continued:

> We demand the city of Göttingen to take measures and provide free hotel rooms for residents of Groner Landstraße 9a-c who wish to do so. People who are not infected with Corona have to be treated like people not infected in any other housing unit. Corona cannot be an excuse to justify repressive measures such as checking the residents' visa status or inquiring about their drug use. Open the hotels now! Open the borders now! (*my translation*)

This all came to me as quite a surprise. Having been in Göttingen for almost a year now, rarely has the city given me the impression of an activist hotspot. The situation, I am told, was different when the punk scene was active in the 1980s, with anti-Nazi demonstrations taking place daily. And Göttingen does have a history of activism as it was one of the major cities of anti-monarchist rebellion in mid-19th century after a group of scholars (including the famous folklorists, Brothers Grimm) known as the Göttingen Seven were kicked out of their posts at the

© 2020 The Authors. City & Society published by Wiley Periodicals LLC on behalf of American Anthropological Association. This is an open access article under the terms of the Creative Commons Attribution License, which permits use, distribution and reproduction in any medium, provided the original work is properly cited. DOI: 10.1111/ciso.12336.

university for defying the King’s orders. Today, activist circles are still somewhat visible but more niche, encompassing those elements of German society who attend leftist meetings organized in squatter venues and subculture cafés. Yet, one can see fragments of activism especially following the tragic murder of George Floyd, which led to Black Lives Matter demonstrations in Göttingen. Today, the city’s foremost squatter house, the Juzi, is adorned with a BLM graffiti that pays homage to George Floyd.

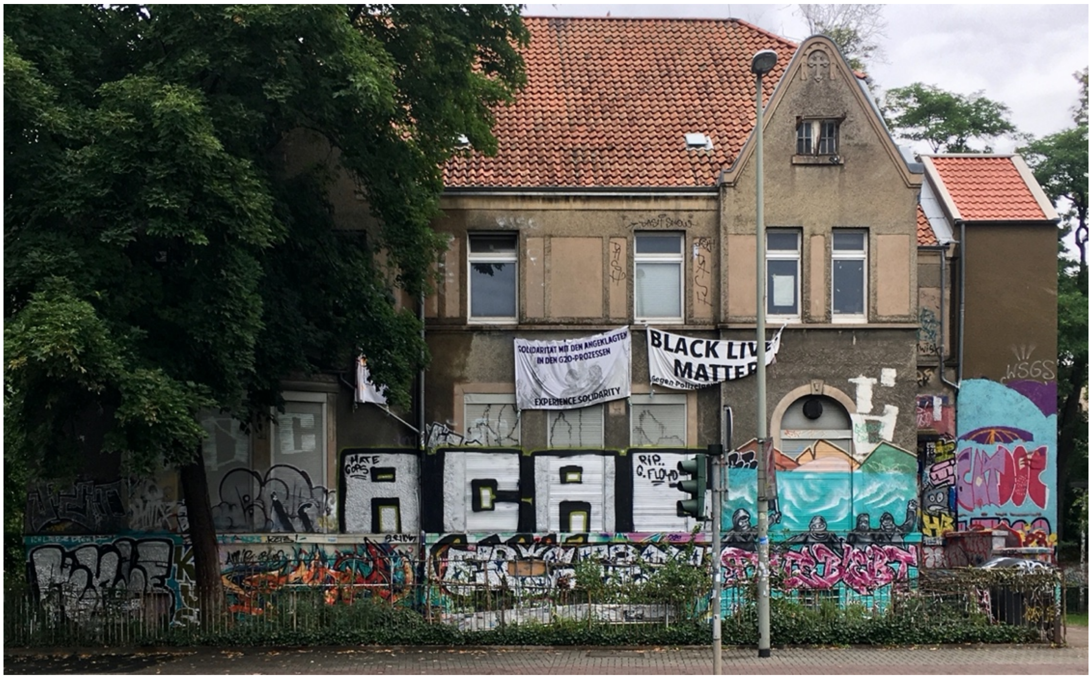

Front façade of Juzi. 2 August 2020. Photo Credit: Oğuz Alyanak

Of all cities in Germany, Göttingen would be my last pick for an urban crisis, especially one that addresses the plight of the urban poor and migrant communities. It is not that these communities do not exist in Göttingen, but they constitute only a fragment of the population. Göttingen is not your typical industrial German city with crowded high-rises that house migrant laborers, refugees, and asylum seekers. Those who have heard of this city of 120,000 probably know it as a calm university town, “die Stadt, die Wissen schafft,” the city that produces knowledge as a

© 2020 The Authors. City & Society published by Wiley Periodicals LLC on behalf of American Anthropological Association. This is an open access article under the terms of the Creative Commons Attribution License, which permits use, distribution and reproduction in any medium, provided the original work is properly cited. DOI: 10.1111/ciso.12336.

third of its population is made up of students, graduate workers, and administrative staff. Hence, it was quite a surprise when Göttingen made it to international news in mid-June, and not just once, but on two accounts. Both were related to the pandemic, and have to do with virus outbreaks in the city's two prominent high rises.

*The Corona High-Rises*

The first case took place in late May in a housing complex known as Iduna-Zentrum. An 18-story apartment which hosts approximately 700 residents living across 407 flats, Iduna was originally built in 1975 as a "Prestige-bau," that is, a luxury complex with balconies, a swimming pool and two bridges connecting it to the university's main campus and the city center. It was even featured in postcards, which displayed it as if to attest to the city's luxurious housing opportunities. Iduna was initially owned by the Iduna Gruppe, a private insurance company, who, over time, sold the flats to private owners. Today, the high-rise has over 200 landlords who rent the rooms to the city, which, in return, rents them out to the recipients of Hartz IV, the lowest level of unemployed benefit offered by the German state. Initially built to accommodate the young scholars attending the University of Göttingen, the high-rise became a residence known for its low-income residents. In a lengthy piece published in the German weekly, *Stern*, it is referred as "das Hotel zur lockeren Schraube," the hotel for people who have loose screws (Stern 2019).

© 2020 The Authors. City & Society published by Wiley Periodicals LLC on behalf of American Anthropological Association. This is an open access article under the terms of the Creative Commons Attribution License, which permits use, distribution and reproduction in any medium, provided the original work is properly cited. DOI: 10.1111/ciso.12336.

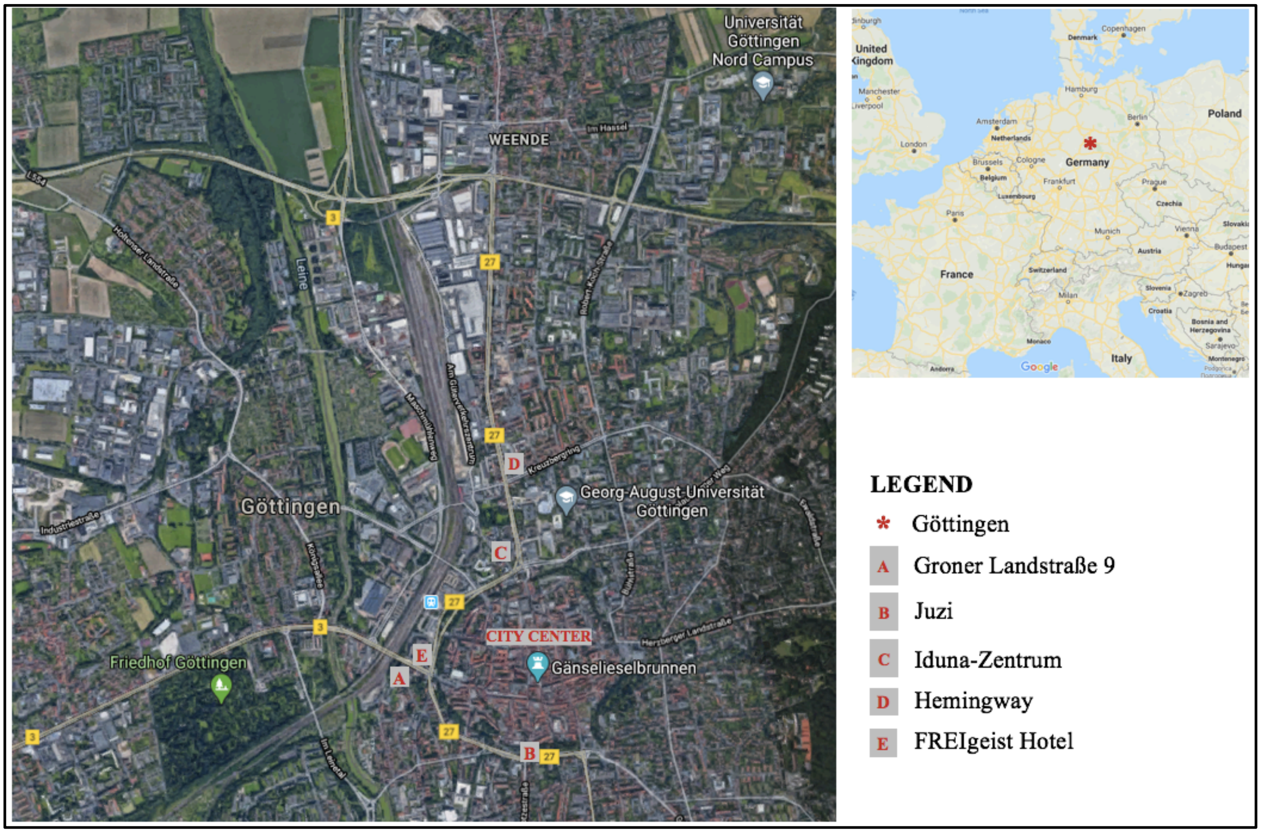

I first heard of Iduna in Hemingway, a *kneipe* (bar) in Iduna's vicinity, which was also a few hundred meters from my flat. My drinking buddy, an ex-punk German man in his early 50s, has told me of its horrendous living conditions—broken or unlocked doors, dirty mattresses, accumulating garbage, and prevalence of meth and flakka users—and advised me against renting a flat there. I later learned that some students did actually live there. And their narratives seemed to corroborate the stories I listened to in the kneipe. Elisabeth, a graduate student, moved into Iduna in early March primarily due to its low rent (500 Euros per month). Her flat was clean, but the outer hallways were dirty. She was not bothered by groups of people—mostly foreign children—gathering in the apartment front to play games. But when she found out that one of the

© 2020 The Authors. City & Society published by Wiley Periodicals LLC on behalf of American Anthropological Association. This is an open access article under the terms of the Creative Commons Attribution License, which permits use, distribution and reproduction in any medium, provided the original work is properly cited. DOI: 10.1111/ciso.12336.

flats on her floor served as the headquarters for what she thinks was a drug-trafficking ring, she was worried.

A few months after she moved in, the first case of COVID-19 was spotted in the high-rise. In the last week of May, one of its residents, a middle-aged resident of Albanian origin, gathered with 30 friends in a shisha bar for the Ramadan festivities (Tagesspiel 2020). When he returned to his flat, he infected 60 others. The housing complex was put under lockdown a few days later, making a name as a “Corona-Hochhaus,” a Corona high-rise (Stern 2020). A day before the police secured the perimeter, Elisabeth had evacuated her flat per her department’s orders, and was forced to take a COVID-19 test (for which she tested negative).

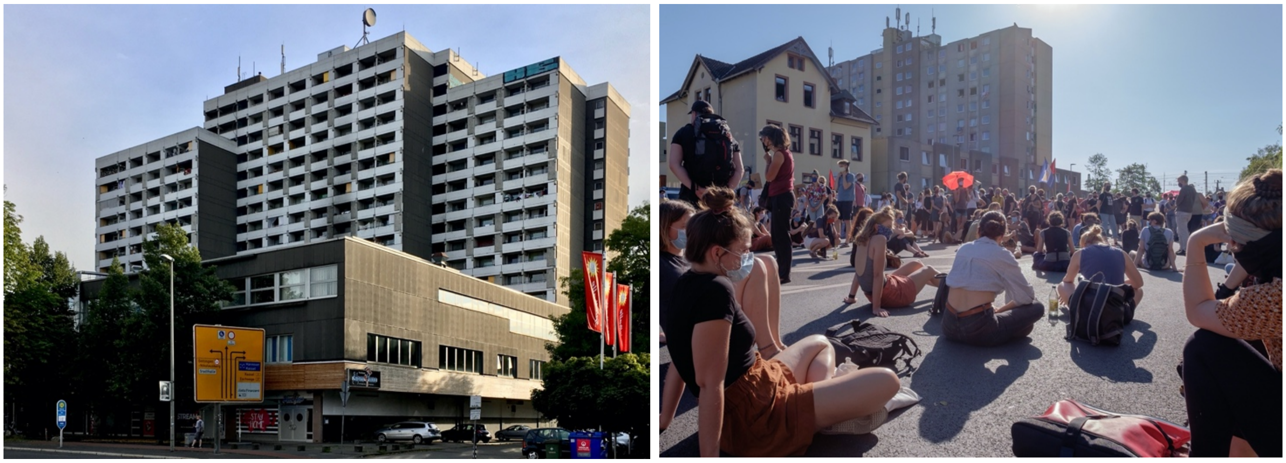

Left: Iduna-Zentrum, pictured from Weender Landstraße. 3 August 2020.
Right: Groner Landstraße 9 (high-rise to the back), pictured from Groner Landstraße the day of the demonstration. Photo Credit: Oğuz Alyanak

The second case took place in Groner Landstraße 9. Although this high-rise lies close to the city center, it took me a few months to come across it. I encountered it during one of my walks around the city with a German colleague. Its grey façade was made up of several hundred windows stacked on top of each other. Most were open, and had laundry hanging from them—an

© 2020 The Authors. City & Society published by Wiley Periodicals LLC on behalf of American Anthropological Association. This is an open access article under the terms of the Creative Commons Attribution License, which permits use, distribution and reproduction in any medium, provided the original work is properly cited. DOI: 10.1111/ciso.12336.

unlikely scene for German residences, but a likely one for migrant dwellings such as this one, where apartments are at a maximum 37 square meters, and occupied by families with several children. My friend described it as “the worst place in Göttingen.” “Iduna,” she asserted, “at least has balconies.”

Like Iduna, Groner Landstraße 9 was put under isolation after the first case of COVID-19 was discovered. But unlike Iduna, the residents of Groner Landstraße 9 fought against the isolation measures, which led to the demonstration a few days later. Up until then, I was not aware of a housing crisis in Göttingen, other than having heard students complain about not finding affordable housing options. Nor was I aware of the city’s urban poor or the precarious living conditions that the demonstrators brought to my attention. The story, as I came to realize, was a typical urban transformation tale, where affordable housing options were cut down to make room for fancy hotels. The city capitalized on one of its main assets—tourism—which came at the expense of those already living in precarious conditions. Why was not the money allocated to them, and instead put into new infrastructural projects? That this question was raised amid a pandemic was further intriguing, helping me realize that COVID-19 was not just a public health crisis, but also an urban crisis. In an attempt to overcome my own ignorance, I grabbed my notebook and camera, and headed to Groner Landstraße.

© 2020 The Authors. City & Society published by Wiley Periodicals LLC on behalf of American Anthropological Association. This is an open access article under the terms of the Creative Commons Attribution License, which permits use, distribution and reproduction in any medium, provided the original work is properly cited. DOI: 10.1111/ciso.12336.

*1-3-1-2*

I arrived at the intersection of Groner Landstraße and Berliner Straße on 23 June at 5 pm. There were already hundreds of demonstrators—some carrying banners ("Stop racist and classicist violence," "Make the rich pay for COVID19") and others wearing shirts with slogans ("No human is illegal") waiting in groups and in the tents set up by various leftist organizations—who had gathered in front of Göttingen's recently built hotel, FREIgeist. One of the demands of this protest was to open the hotels for residents living in situations similar to the Groner Landstraße. There are at least 1300 free rooms in hotels across Göttingen, the Anarchists of Göttingen had mentioned in a social media post. Thus, our gathering spot was a statement in itself.

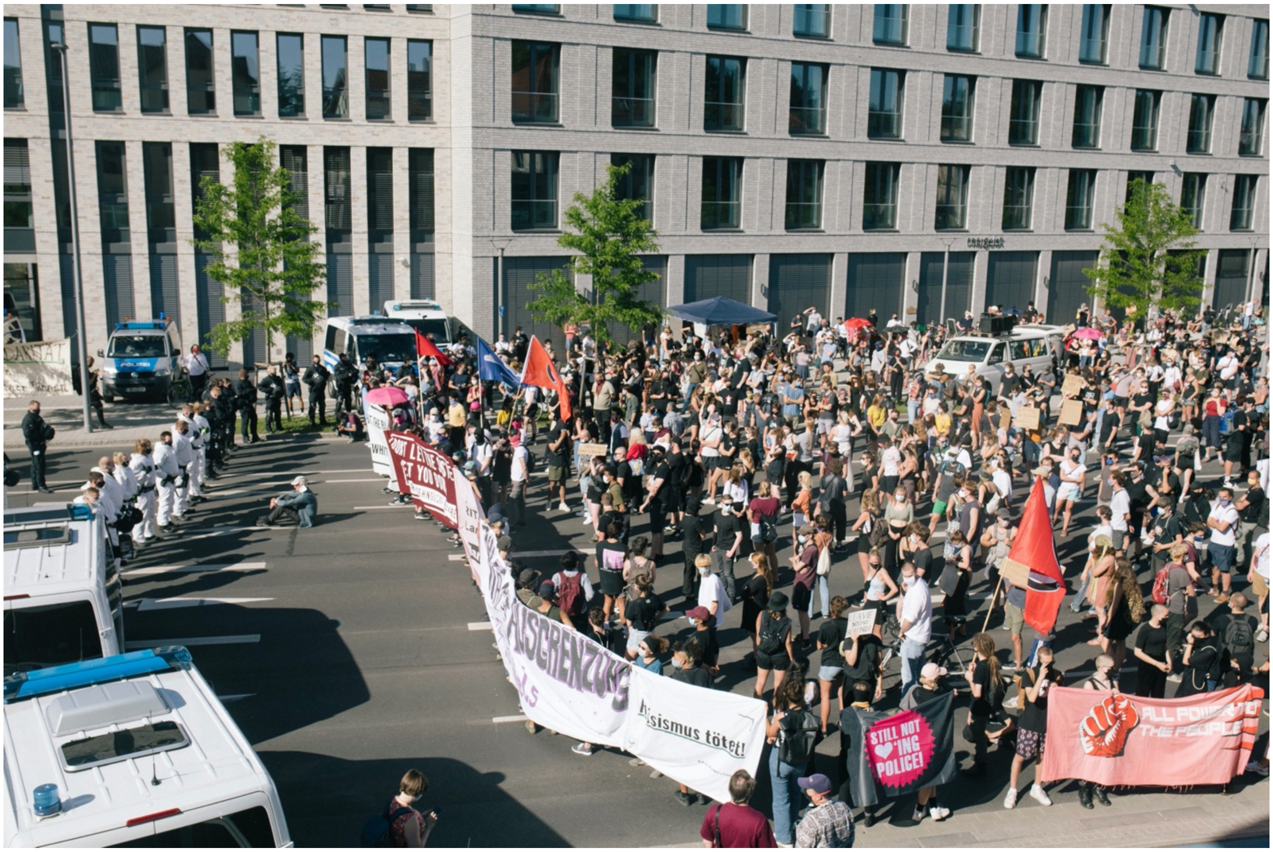

Groner Landstraße demonstration. 23 June 2020. Photo Credit: Michael Trammer

© 2020 The Authors. City & Society published by Wiley Periodicals LLC on behalf of American Anthropological Association. This is an open access article under the terms of the Creative Commons Attribution License, which permits use, distribution and reproduction in any medium, provided the original work is properly cited. DOI: 10.1111/ciso.12336.

Soon after, music started to blast from the loudspeakers set up on a van a few meters before the police barrier.  "1-3-1-2. Fick die Polizei. Mittelfinger high. Die ganze clique ist mit dabei," sang the Kurdish-German hip hop artist, Ebow. Fuck the police, the whole gang is here with their middle fingers up in the air.

There we were. The 400 or so protestors who had gathered by FREIGeist were enough of a presence to force the police to cut off traffic. Slowly, we moved towards the high rise, which was surrounded by metal fences and patrolled by the German police and their dogs, which barked at us every time we clapped hands. The street was cut off by a line of police forces wearing white hazmat suits and protective gear. Facing them was a string of protestors holding Antifa flags and large banners, some in German and others in English. They read: "Solidarity against Exclusion #openthehotels," "Solidarity with Groner Landstraße 9," "Don't Let the System Get You Down," "Fight the Racist State, Its Cops, and White Supremacy," "Racism Kills," and "Still not Loving the Police." They remained there until the demonstration came to an end.

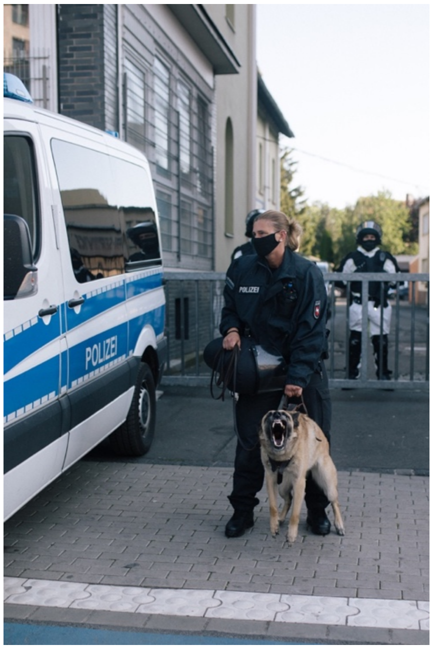

Groner Landstraße demonstration.
23 June 2020.
Photo Credit: Michael Trammer

© 2020 The Authors. City & Society published by Wiley Periodicals LLC on behalf of American Anthropological Association. This is an open access article under the terms of the Creative Commons Attribution License, which permits use, distribution and reproduction in any medium, provided the original work is properly cited. DOI: 10.1111/ciso.12336.

As each demonstrator found a spot on the asphalt and by the sidewalk, talking to each other or watching the curious faces who were staring at us from the windows of Groner Landstraße 9a-c, one of the organizers shouted into the microphone: "Hello Groner Landstraße 9a-c. Hello demonstrators!"

After a few brief remarks explaining the purpose of the demonstration, and clarifying that the organizers had intended to gather in front of the Sparkasse bank by the high-rise—the city forbid them from doing so—they shared a cautionary remark. "There may be civil police among us. In case you need legal help, please call the following number: 05517708000." Given that Antifa organizations would be in attendance, and eight police officers were injured in the clash two days prior, friends had warned me that things could get violent. The demonstration, however, proved to be anything but violent. In fact, with the exception of two slogans—"Alle zusammen gegen den Faschismus" (All together against fascism), which was chanted as a representative from the Free Workers Union of Göttingen took the microphone, and "Fa, anti, antikapitaliste, fa, anti, antikapitaliste," following the speech by an Antifa representative—the crowd was calm.

The demonstration continued with a representative from the city's Roma center, who briefed us on the Center's position on the lockdown. Given that a large number of residents of the high-rise were of Roma background, the representative was a key player. His speech focused on the struggles of the Roma people in obtaining accommodation with better living conditions and highlighting the precarity of foreigners in Germany as evident in "Corona Landstraße." His depiction of the high-rise as a Corona hotspot was apt given the conditions in the high-rise—a point that became clear as we listened to pre-recorded interviews with residents. "Crowded, pest

© 2020 The Authors. City & Society published by Wiley Periodicals LLC on behalf of American Anthropological Association. This is an open access article under the terms of the Creative Commons Attribution License, which permits use, distribution and reproduction in any medium, provided the original work is properly cited. DOI: 10.1111/ciso.12336.

infested small flats with tiny corridors," reiterated one of the organizers after the recorded interview ended. She demanded the city to move its residents to a hotel free of charge. "No social distancing is possible inside this house," she argued, as the apartments were at a maximum 37 square meters, and the smaller flats a mere 19 square meters. "600 people, among them 200 children, had no food when they were first put on lockdown" she asserted, and added: "The food packages provided included no special diet for children. They live in precarity."

But precarity, as the next speaker made clear, was not the result of the pandemic. It was a structural condition that has long been there and perpetuated strategically both by the media and the state. The speaker reminded us of the xenophobic predisposition of the mediatic focus on shisha bars, which are attended mostly by people of migrant backgrounds, such as the Albanian man from Iduna. These accounts perpetuated racist attitudes already prevalent in Germany. According to a policy paper published by the University of Bamberg, which was mentioned in her speech, immigrants faced segregation in the German housing market, both by being assigned by the state to low-income housing units, and for those who have the means, by not being permitted by the landlords to move into better residences due to their foreigner status (c.f. Rodhe 2009). Housing complexes such as Iduna or Groner Landstraße 9 were therefore not exceptions, but a long-standing reality whose stark presence became further visible during the pandemic.

© 2020 The Authors. City & Society published by Wiley Periodicals LLC on behalf of American Anthropological Association. This is an open access article under the terms of the Creative Commons Attribution License, which permits use, distribution and reproduction in any medium, provided the original work is properly cited. DOI: 10.1111/ciso.12336.

Then came another announcement, this time reminding us that the police would intervene unless we kept our distance from each other as part of the city's Corona mandate. This announcement was followed by boos and chants. A couple of more speeches later, the demonstration came to an end.

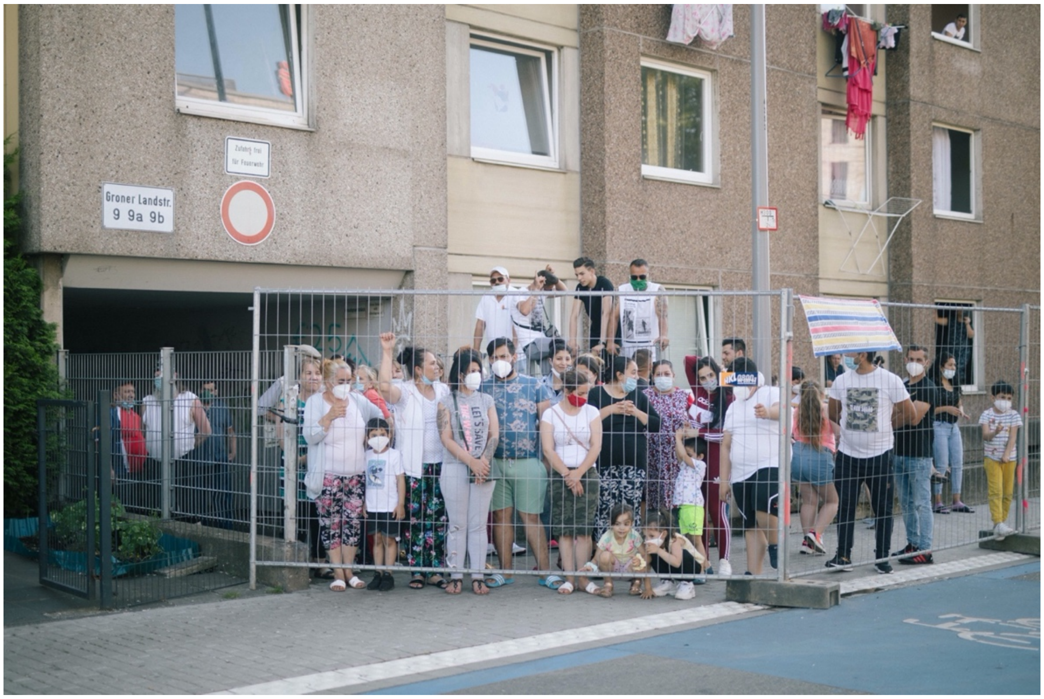

Groner Landstraße demonstration. 23 June 2020. Photo Credit: Michael Trammer

*COVID-19 as a Crisis in Urban Justice*

Lockdown. Quarantine. Stay-at-home. We have familiarized ourselves with these terms in recent months as more than half of the world's population has been requested or forced to stay put amid the pandemic. For those who have the means, staying at or working from home may be a welcomed change of scenery. Yet for others, such as the residents of Iduna or the Groner Landstraße, being stuck in a 30 meter square flat with a family is not a cherished option.

© 2020 The Authors. City & Society published by Wiley Periodicals LLC on behalf of American Anthropological Association. This is an open access article under the terms of the Creative Commons Attribution License, which permits use, distribution and reproduction in any medium, provided the original work is properly cited. DOI: 10.1111/ciso.12336.

The calls to stay home and self-isolate, which, we are told, are necessary measures to slow down the spread of the virus, bring to fore problems with precarious living conditions of immigrants, refugees and asylum-seekers who are usually placed in crumbling housing units. That it is foreigners who have to face the burden, moreover, bring to our attention the underlying racist and xenophobic attitudes, which, to this day, are part and parcel of the German society. On the one hand, there is the long-standing Orientalist fear that the unruly foreigners would not self-isolate, and should therefore be forced to do. And on the other, there is the reality that most of the hotspots, such as the high-rises, or workplaces such as slaughterhouses, are inhabited by foreign/migrant workers, who are living or working in decrepit conditions not out of will, but simply due to lack of better alternatives.

COVID-19 is a not just crisis in public health. As the Groner Landstraße demonstration makes clear, it also makes visible problems pertaining to urban life, which have as much to do with urban justice as with racialized accounts dominating public conversations.

**Acknowledgements:**
The author would like to thank Matthew Abel for his careful reading of earlier drafts of this article, and Michael Trammer for providing the visuals from the Groner Landstraße demonstration. Open access funding enabled and organized by Projekt DEAL.

© 2020 The Authors. City & Society published by Wiley Periodicals LLC on behalf of American Anthropological Association. This is an open access article under the terms of the Creative Commons Attribution License, which permits use, distribution and reproduction in any medium, provided the original work is properly cited. DOI: 10.1111/ciso.12336.

**References**

Eddy, Melissa. 2020. “Flare-Up in Virus Cases Sets Back Germany’s Efforts to Reopen.” *New York Times*. 25 June. https://www.nytimes.com/2020/06/25/world/europe/germany-coronavirus-reopening.html. Last accessed August 1, 2020.

Posaner, Joshua. 2020. “Germany reckons with second wave risk.” *Politico*. 23 June. https://www.politico.com/news/2020/06/23/germany-reckons-with-second-wave-risk-336077. Last accessed August 1, 2020.

Rohde, Caterina. 2009. “Positioning of Migrants into the Housing Market: Structural Conditions and Urban Policies.” Europaische Forum fur Migrationstudien Papers 1: 1-56.

Saller, Joseph. 2019. “Hartz IV, Drogen, geplatzte Träume: Begegnung mit den Mietern eines Göttinger Hochhauses,” *Stern*. 12 May. https://www.stern.de/panorama/gesellschaft/goettingen--begegnung-mit-mietern-des-iduna-zentrums-8704080.html. Last accessed August 1, 2020.

Martyr, Kate. 2020. “Germany: Quarantined housing block residents attack police.” *Deutsche Welle*. 20 June. https://www.dw.com/en/germany-quarantined-housing-block-residents-attack-police/a-53885044. Last accessed August 1, 2020.

“Erneuter Corona-Ausbruch in Göttinger Hochhaus: 700 Leute unter Quarantäne.” *Stern*. 18 June. https://www.stern.de/gesundheit/weiterer-fall-in-goettingen--hochhaus-mit-700-leuten-unter-quarantaene-9305824.html. Last accessed August 1, 2020.

“Was der Corona-Ausbruch in der Stadt über soziale Spaltung verrät.” *Der Tagesspiel*. 11 June 2020. https://www.tagesspiegel.de/themen/reportage/die-elenden-von-goettingen-was-der-corona-ausbruch-in-der-stadt-ueber-soziale-spaltung-verraet/25905638.html. Last accessed August 1, 2020.

© 2020 The Authors. City & Society published by Wiley Periodicals LLC on behalf of American Anthropological Association. This is an open access article under the terms of the Creative Commons Attribution License, which permits use, distribution and reproduction in any medium, provided the original work is properly cited. DOI: 10.1111/ciso.12336.
